# Scholarly productivity of faculty in primary care roles related to tenure versus non-tenure tracks

**DOI:** 10.1186/s12909-020-02085-6

**Published:** 2020-05-29

**Authors:** Michaela M. Braxton, Jhojana L. Infante Linares, Dmitry Tumin, Kendall M. Campbell

**Affiliations:** 1 Master of Social Work Student, College of Health and Human Performance, East Carolina University, Greenville, NC USA; 2 Office of Data Analysis and Strategy, Brody School of Medicine, East Carolina University, Greenville, NC USA; 3 Department of Pediatrics, Brody School of Medicine, East Carolina University, Greenville, NC USA; 4 Research Group for Underrepresented Minorities in Academic Medicine, Division of Academic Affairs, Brody School of Medicine, East Carolina University, 600 Moye Blvd AD-47, Greenville, NC 27834 USA

**Keywords:** Faculty track, Primary care, Scholarly productivity

## Abstract

**Background:**

Increasing the number of primary care physicians is critical to overcoming the shortage of healthcare providers. Primary care physicians are increasingly called upon to address not only medical concerns but also behavioral health needs and social determinants of health which requires ongoing research and innovation. This paper evaluated scholarly productivity of faculty in tenure versus non-tenure tracks in primary care roles, defined as family medicine, internal medicine, internal medicine/pediatrics and pediatrics.

**Methods:**

The study included physician faculty in the clinical departments of Brody School of Medicine serving between the 2014–2015 and 2018–2019 academic years. Department, track, and rank at the beginning of each academic year (e.g., 2014–2015) were correlated with having any publications in the following calendar year (e.g., 2015), as determined from a search of the Scopus database.

**Results:**

A total of 1620 observations and 542 unique faculty were included in the analysis. As of 2018–2019, 19% percent of primary care faculty were either tenured or on tenure track, as compared to 41% of faculty in other departments (*p* < 0.001). Primary care departments were also disproportionately staffed by junior faculty (60% as compared to 48% in other departments; *p* = 0.087). The proportion of faculty with any publications was significantly higher for faculty on the tenure track compared to those not on the tenure track (34% vs. 14%, *p* < 0.001).

**Conclusions:**

Academic productivity was lower among non-tenure-track physician faculty, as measured by publication in peer-reviewed journals. This was exacerbated among faculty in primary care departments, who were also more likely to hold non-tenure-track appointments. The loss of tenure-track positions disproportionately impacts scholarly activity in primary care and may be limiting progress in care-oriented research. Findings suggest that providing non-tenure faculty the time and resources to be involved in research, in addition to their clinical work, as well as access to research collaborators and mentors can promote scholarly activity among this group.

## Background

Increasing the number of primary care physicians is critical to overcoming the shortage of health care providers in clinics and hospitals [1]. Primary care physicians are increasingly called upon to address not only patients’ medical concerns but also behavioral health needs and social determinants of health. Training primary care physicians equipped to handle these needs has been a key aim of community-driven, primary care-focused medical schools, but data have shown that these new schools are not producing any more primary care physicians than older, more traditional medical schools [2]. A further limitation of this need-driven approach focused on the provision of clinical care is a potential lack of attention to physicians who engage in primary care research that would advance the field and disseminate innovations in practice. Primary care physicians hired into positions that are focused on clinical care (i.e., non-tenure track) may be too overwhelmed with the growing needs of clinical care to participate in increasing scholarship in this area. Consequently, advances in primary care are likely to remain under-studied and localized, rather than being disseminated through scholarly channels.

Despite the contributions of academic researchers from other disciplines, physician leadership is needed in primary care research, since physicians practicing in this area have been leaders in innovative care discoveries and see firsthand the consequences of social and economic changes impacting community health [3, 4]. Physician abandonment of this work mostly to non-physician colleagues can create gaps in methodology and omission of the physician’s voice. This abandonment is often not by choice but can be dictated by the clinical demands of the local community. Increased clinical demand can pull the primary care physician with an interest in research to only providing clinical care. Compounded by the need to generate clinical revenue in a fee for service healthcare model, the physician-researcher can become extinct in primary care specialties. Conversely, research-minded primary care physicians can add perspective to care-oriented research as it relates to day-to-day patient care experiences, thus strengthening the methodology and utility of this research.

At academic medical centers, the disappearance of tenure for primary care physicians has compounded the barriers to their engagement in care-oriented research. The drive to achieve tenure motivates curiosity, stimulates ideas and engages the primary care physician beyond a care provision only model. Tenured positions are often associated with job security and academic freedom [5]. Tenure is also associated with leadership in medicine and may be a requirement for opportunities such as research grants and eligibility for professional awards [6]. The number of faculty with tenure and in tenure earning tracks has decreased [7, 8]. This decrease has disproportionately impacted underrepresented minority faculty members and women in academic medicine [6, 9]. While prior research has considered the impact of tenure-track appointment on the scholarly productivity of faculty at academic medical centers, it is unclear how tenure appointments impact primary care physician research [10, 11]. Therefore, in this pilot study, we analyzed the impact of faculty track and presence or absence of tenure on physician faculty members’ scholarly publication at a rural, community-based medical school.

## Methods

The study was deemed not to involve human subjects research by the Institutional Review Board at East Carolina University (ECU). The Brody School of Medicine (BSOM) at ECU is located in a rural region in eastern North Carolina and employs over 400 faculty. The study included physician faculty members with at least 50% full-time equivalent (FTE) appointments in the clinical departments of BSOM serving between the 2014–2015 and 2018–2019 academic years. Faculty with primary appointments in BSOM administration were excluded, as well as faculty in basic science departments and research faculty appointed in clinical departments. Faculty rosters at the start of each academic year (July) were obtained from BSOM Human Resources (HR). Rank and publication data were analyzed for each faculty member year-by-year, with rank at the beginning of the academic year (e.g., July 2018 for Academic Year 2018–2019) matched to the number of publications in the following calendar year (in this case, 2019).

BSOM faculty appointments include tenured and tenure-track positions, as well as fixed-term positions that are ineligible for tenure (clinical track). Policies regarding appointment and promotion on each track are set by individual departments and reviewed at least every 10 years. Assistant professor and clinical instructor positions were considered junior ranks, while the associate professor and professor positions were considered to be of senior rank. For each year of data, faculty members’ track and rank were determined as of the beginning of the academic year and faculty members’ percent FTE in each academic year was queried. From the 2017–2018 academic year onwards, faculty rosters also included self-identified gender and race (grouped for analysis as Black or African American, White, Asian, and Other or not reported). Faculty were classified as primary care physicians if their primary appointment was in Family Medicine, General Pediatrics (including Adolescent Medicine), Internal Medicine-Pediatrics, or General Internal Medicine. This study was conducted in the United States (US), where the generalist specialties of internal medicine and pediatrics are considered to be primary care specialties. Such designations may differ outside of the US. Other departments and divisions, including subspecialties in internal medicine and pediatrics, served as the comparison group.

The primary outcome was the annual number of scientific publications. Publication data were obtained from the Scopus database by cross-referencing faculty rosters with a list of publications authored by ECU affiliates. The affiliation search included the keywords “Brody,” “BSOM,” “East Carolina,” “ECU,” and variations on the name of the associated teaching hospital: “Vidant,” “VMC,” “Pitt County Memorial,” and “PCMH.” Eligible publications included original scientific articles, research notes, reviews, and surveys. Other publication types, such as conference abstracts and letters to the editor were excluded. After matching publication data to BSOM faculty data, publication counts were manually checked for authors with the same last name and first initial, and for authors with a high number of publications (>99th percentile) in a given year. Publication data were queried for calendar years 2015 to 2019, with data collection concluding on January 9, 2020.

Faculty characteristics and study outcomes were summarized as counts with percentages or medians with interquartile ranges. As many faculty did not have publications during the study period, the authors focused on a dichotomized variable of publication in a given year (any vs. none) for multivariable analysis of annual publication outcomes. The association between independent variables and the outcome of having any publications in a given year was summarized as an odds ratio (OR) with a 95% confidence interval (CI) in the multivariable analysis, which included one observation for each year that a faculty member was employed at our institution. The authors fit an initial multivariable model including year, track, rank, % FTE, and specialty (primary care vs. other departments) for the overall study period (academic years 2014–2015 through 2018–2019). The authors then fit a second multivariable model adding gender and race controls to the academic years in which these data were available (2017–2018 and 2018–2019). In each time period, the impact of faculty track was compared between primary care and other departments by adding an interaction between these factors to the model. Standard errors were adjusted for the clustering of multiple observations within faculty, accounting for non-independence of publication outcomes in different years for the same faculty member. Data analysis was performed in Stata/SE 15.1 (College Station, TX: StataCorp, LP) and *P* < 0.05 was considered statistically significant.

## Results

A total of 1620 observations (one for each faculty and academic year) and 542 unique faculty were included in the analysis. Eleven clinical departments were represented, with departmental size and proportion of faculty on the tenure track summarized for the 2018–2019 academic year in Table 1 (Internal Medicine and Pediatrics were each split between primary care and subspecialist faculty). Nineteen percent of primary care faculty were either tenured or on the tenure track, as compared to 41% of faculty in other departments (*p* < 0.001). Primary care departments were also disproportionately staffed by junior faculty (60% as compared to 48% in other departments; *p* = 0.087).

**Table 1:** Characteristics of clinical departments included in the analysis

Department	Number of clinical faculty^**a**^	Percent of junior faculty^**a**^	Percent of faculty on tenure track^**a**^
***Primary care departments***	**70**	**60**	**19**
Internal Medicine (General)	19	79	0
Family Medicine	32	63	19
Pediatrics (General)	19	37	37
***Other departments***	**266**	**49**	**41**
Emergency Medicine	46	80	22
Rehabilitation Medicine	12	42	25
Radiation Oncology	3	33	33
Pediatrics (Subspecialty)	43	40	35
Pathology	17	35	35
Internal Medicine (Subspecialty)	59	54	36
Psychiatry and Behavioral Medicine	16	44	38
Obstetrics and Gynecology	14	36	43
Surgery	28	36	64
Cardiovascular Sciences	28	32	86

^a^ At the beginning of the 2018–2019 academic year

**Fig. 1:**
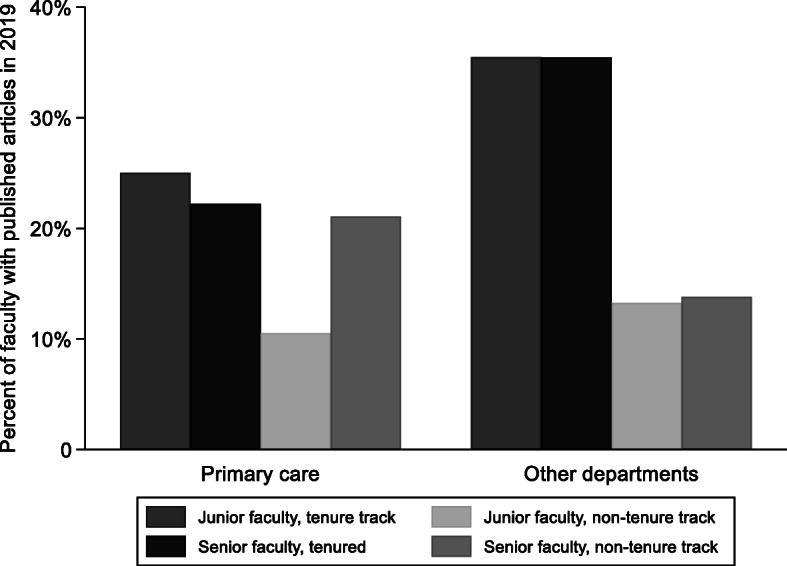
Percentage of physician faculty employed at the start of the 2018–2019 academic year with at least 1 publication in 2019, by specialty, track, and rank

Other characteristics of 336 faculty members employed at the start of the 2018–2019 academic year are summarized in Table 2, comparing faculty in primary care to other departments. Overall, 21% of faculty employed at the start of the 2018–2019 academic year had at least one publication in the calendar year 2019. The proportion of faculty with any publications was significantly higher for faculty on the tenure track compared to those not on the tenure track (34% vs. 14%, *p* < 0.001). A further breakdown by department, track, and rank is shown in Fig. 1. Junior, non-tenure track primary care faculty were least likely to have any publications in 2019 (11%), while tenure-track faculty in other departments were most likely to have any publications (35% each for junior and senior faculty in these roles). A sub-analysis of faculty in primary care departments, comparing Family Medicine to General Pediatrics and General Internal Medicine, found that among 32 faculty in Family Medicine, 4 (13%) had at least 1 publication in 2019; compared to 7 of 38 (18%) of faculty in other primary care departments (Fisher’s exact test *p* = 0.533).

**Table 2:** Characteristics of clinical faculty at the beginning of the 2018–2019 academic year, comparing primary care to other departments

Variable	Primary care (***N*** = 70)	Other departments (***N*** = 266)	P
	N (%) or median (IQR)	N (%) or median (IQR)	
Academic track and rank			
Junior faculty	42 (60%)	129 (49%)	0.087
Tenured or tenure-track	13 (19%)	110 (41%)	< 0.001
FTE (percent)	100(100, 100)	100(100, 100)	0.233
Gender			0.146
Male	34 (48%)	155 (58%)	
Female	36 (51%)	111 (42%)	
Race			0.688
Black or African American	4 (6%)	16 (6%)	
White	44 (63%)	164 (62%)	
Asian or Asian American	11 (16%)	31 (12%)	
Other or not reported	11 (16%)	55 (21%)	

*IQR* interquartile range, *FTE* full time equivalent

Multivariable regression results for the entire study period are summarized in Table 3. The strongest predictor of having any publications in a given year was an appointment to a tenured or tenure-track position (OR = 3.95; 95% CI: 2.74, 5.69; *p* < 0.001). After controlling for faculty track, primary care positions were no longer associated with a statistically significant disadvantage in the likelihood of having any publications in a given year (OR = 0.70; 95% CI: 0.40, 1.23; *p* = 0.217). After adjusting for gender and race in the two most recent academic years (Table 4), we found that tenure-track faculty remained significantly more likely to have at least one publication in a given year (OR = 3.68; 95% CI: 2.27, 5.99; *p* < 0.001). Females were 40% less likely than males to have any publication in a given year (OR = 0.60; 95% CI: 0.37, 0.96; *p* = 0.035), controlling for faculty track, rank, department (primary care vs. other departments), and %FTE. Faculty identifying as Asian or Asian Americans were more likely than faculty identifying as White to have published at least one article in a given year (OR = 2.00; 95% CI: 1.08, 3.71; *p* = 0.028).

**Table 3:** Logistic regression of the annual number of publications in 2015–2019 (*N* = 1620 observations)

Independent variable	OR	95% CI	P
Academic year	1.05	0.96, 1.15	0.291
Tenure track	3.95	2.74, 5.69	< 0.001
Primary care	0.70	0.40, 1.23	0.217
Junior rank	0.99	0.69, 1.42	0.971
FTE % x10^a^	1.19	0.98, 1.44	0.079

*CI* confidence interval, *IRR* incidence rate ratio, *OR* odds ratio

^a^ Estimated effect represents the change in the outcome associated with a 10-point increase in this percentage

**Table 4:** Logistic regression of the annual number of publications in 2018–2019 (*N* = 653 observations)

Independent variable	OR	95% CI	P
Academic year	0.97	0.69, 1.34	0.834
Tenure track	3.68	2.27, 5.99	< 0.001
Primary care	0.88	0.48, 1.64	0.698
Junior rank	1.04	0.64, 1.70	0.863
FTE % x10^a^	1.15	0.90, 1.47	0.258
Gender			
Men	Ref.		
Women	0.60	0.37, 0.96	0.035
Race			
Black or African American	1.56	0.55, 4.39	0.402
White	Ref.		
Asian or Asian American	2.00	1.08, 3.71	0.028
Other or not reported	1.18	0.67, 2.05	0.569

*CI* confidence interval, *IRR* incidence rate ratio, *OR* odds ratio

^a^ Estimated effect represents the change in the outcome associated with a 10-point increase in this percentage

## Discussion

Primary care physicians at academic medical centers face increased pressure to focus on clinical care, especially addressing the complex needs of medically underserved populations, to the exclusion of scholarly work developing and disseminating innovations in care delivery. This is compounded by the increased appointment of primary care physicians to non-tenure track positions, where they receive less support for research and encounter lower expectations for scholarly productivity. At a rural, community-based academic medical center, the authors found lower academic productivity among clinical physician faculty, as defined by publication in peer-reviewed journals. This was exacerbated among faculty in primary care departments, and especially among faculty appointed to non-tenure track positions, which were far more common in primary care departments. Thus, the loss of tenure-track positions disproportionately impacts scholarly activity in primary care and may be limiting progress in care-oriented research.

Tenure for the academic physician fits into a research infrastructure that affords a faculty member freedom of inquiry, the ability to test hypotheses, and a scientific approach to questioning the status quo and current processes. Reasons for decreasing numbers of tenure track positions can include the thinking that many who achieve the rank of tenure become unproductive and that increasing clinical demands at academic medical centers make tenure less relevant. Yet, the loss of tenure carries profound negative effects on faculty development at medical schools. Faculty in nontenured positions have a lower likelihood of promotion, higher likelihood of attrition, lower salaries and fewer leadership opportunities [12, 13]. Additionally, clinical track faculty have reduced scholarly productivity as compared to tenure track faculty [10, 11], lower expectations, limited supported time for scholarship and other limitations which could negatively impact innovations and discoveries, especially in primary care.

Prior research on the scholarly productivity of physician faculty at academic medical centers has focused on the impact of academic rank, not faculty track. For example, a recent systematic review and meta-analysis by Zaorsky and colleagues included only faculty rank and not faculty track as a predictor of publication productivity [14]. While some studies have combined data from clinical track and tenure track faculty, other studies have excluded faculty on the clinical track altogether [15]. This omission can obscure the extent to which appointments off the tenure track can hinder faculty productivity, especially in primary care fields. This study demonstrates that non-tenure track junior faculty in primary care have the lowest levels of scholarly productivity, with only 11% of faculty in this group publishing any peer-reviewed articles in the most recent year of the study’s data. Primary care departments also had lower proportions of faculty on the tenure track, and on multivariable analysis, tenure emerged as the strongest predictor of scholarly productivity, while faculty rank was not independently associated with this outcome. These data are consistent with the work of Xierali et al. who found a threefold drop in tenured or tenure track Family Medicine physicians from 1977 to 2017 [16], and Walling et al., who found that Family Medicine physicians were least likely among full-time faculty at US medical schools to be in a tenured or tenure-track appointment [17].

The loss of tenure-track positions in primary care appears to reduce opportunities for scholarly activity for individual faculty in these departments. Moreover, at medical schools with a primary care focus, this barrier to scholarly productivity can lead to institution-wide repercussions, with limited opportunities for scholarly project collaborations, reduced expectations for scholarly activity, a shortage of academic mentors, and limited opportunities for junior faculty to build a national reputation and demonstrate leadership in academic medicine. Prior research has documented that coauthorship and mentorship networks are critical for junior faculty success and the likelihood of promotion [18–20]. Particularly, access to mentors who are central in their field is an important facilitator of publication productivity for faculty in family medicine, pediatrics, general internal medicine, and internal medicine/pediatrics [19]. In a qualitative analysis of Family Medicine departments with successful research programs, Liaw and colleagues also emphasized the importance of junior faculty making connections with senior mentors in their own department, or in national networks [21]. Therefore, loss of tenure track appointments for primary care physicians can have long-standing repercussions on institutions’ ability to support and retain faculty, if successive cohorts of faculty in primary care departments include fewer and fewer faculty members whose role includes a research component and who are able to secure extramural funding for their work.

As National Institutes of Health (NIH) support shifts towards basic science research, faculty members struggle, and primary care scholarship is left by the wayside as departments focus on delivering clinical care in resource-constrained environments [22, 23]. In a longitudinal study of generalists in academic medicine, Blazey-Martin and colleagues found that this group had the fewest publications and was least likely to be promoted, as compared to medical specialists or surgical specialists [24]. Inequality between departments may also be increasing, as federal funding for primary care research has trended towards supporting fewer and more senior investigators [25]. Although some initiatives have explored enhanced training in research skills for physicians in primary care fields [26], only a system-wide investment in primary care research can overcome the structural barriers associated with pursuing this work [27]. As suggested findings of this pilot study, such an initiative should include a strategy of increasing the number of tenure-track appointments in primary care, in addition to providing faculty in these departments with the time and resources they need to pursue scholarly productivity [28]. This study highlights differences among faculty in scholarly activity at a single center, but the differences across institutions are likely even larger. This may profoundly limit the advances in primary care possible through applying a scholarly lens to the practices and innovations of community-based medical schools.

During the years in which faculty demographic data were available (academic years 2017–2018 through 2018–2019), our study revealed several differences in publication based on race/ethnicity as well as gender. As in previous studies of medical school faculty, we found that women were less likely to publish than men, but due to limitations of available data, we were unable to evaluate explanations that have been proposed with regards to differences in work hours or work-life balance [29]. Compared to white faculty, we found that Asian or Asian American faculty were twice as likely to publish at least one article in a given year; however, the difference in publication between Black or African American faculty and white faculty was not statistically significant. Previous multi-institutional research has found that medical school faculty from underrepresented minority (URM) groups, including Black or African American, Hispanic or Latino, Alaskan Native, American Indian, and Native Hawaiian and Other Pacific Island populations, had fewer scholarly publications than white faculty over a 17-year follow-up period [30]. The impact of this disparity within single institutions needs additional study. Exploring racial and ethnic disparities in faculty track and publication is important due to promotion disparities, diversity effort disparities, and other disparities of the minority tax that hinder scholarship for underrepresented minorities [31, 32].

The design of this study introduced both strengths and limitations to the authors’ conclusions. A single-center analysis permitted analyzing individual faculty scholarly outcomes within the context of their faculty track, a measure unavailable in many prior studies [14]. However, this may have limited the generalizability of the findings to other centers. Demographic data were not available on faculty rosters over the entire study period, and were subject to incomplete and voluntary reporting, limiting the ability to examine racial and ethnic disparities in faculty track and publication. The retrospective nature of this study precluded examining how faculty’s individual goals and the mentorship or guidance they had received influenced their scholarly activity. While self-selection into tenure vs. non-tenure tracks can influence faculty members’ decision to pursue scholarly activity, it is important to note that the choice of a tenure-track position is increasingly constrained by the trend of fewer tenure-track positions being available at medical schools. Therefore, future research could analyze the impact of faculty track specifically within a matched cohort of faculty who had expressed comparable intentions of pursuing an academic career while still in training.

The limited year range of this study meant that it lacked statistical power to assess the impact of individual career trajectories (e.g., switching from one track to another, being promoted on the same track). The authors used a limited outcome measure of peer-reviewed journal publication, which did not capture the full gamut of scholarly activities at academic medical centers. Critically, the authors could not assess differences between departments in progress to the publication of scholarly projects that were started by faculty in each department, especially care-oriented research and quality improvement projects. The use of the Scopus database may also have led to under-counting scholarly output in cases where authors’ published names and affiliations were not rendered consistently with the information on the faculty rosters, or for recently published articles not yet indexed in this database.

## Conclusion

Academic productivity was lower among non-tenure-track physician faculty, as measured by publication in peer-reviewed journals. This was exacerbated among faculty in primary care departments, who were also more likely to hold non-tenure-track appointments. The loss of tenure-track positions disproportionately impacts scholarly activity in primary care and may be limiting progress in care-oriented research. Findings suggest that providing non-tenure faculty the time and resources to be involved in research, in addition to their clinical work, as well as access to research collaborators and mentors can promote scholarly activity among this group. Findings also stress the importance of supporting research of all faculty through research support infrastructure, time allocation, funding and staffing to ensure innovation and discovery in primary care.

## Data Availability

All data generated or analyzed during this study are included in this published article.

## Abbreviations

BSOM: Brody School of Medicine
ECU: East Carolina University
VMC: Vidant Medical Center
PCMH: Pitt County Memorial Hospital
